# Annotation of novel neuropeptide precursors in the migratory locust based on transcript screening of a public EST database and mass spectrometry

**DOI:** 10.1186/1471-2164-7-201

**Published:** 2006-08-09

**Authors:** Elke Clynen, Jurgen Huybrechts, Peter Verleyen, Arnold De Loof, Liliane Schoofs

**Affiliations:** 1 Laboratory of Developmental Physiology, Genomics and Proteomics, K.U.Leuven, Naamsestraat 59, B-3000 Leuven, Belgium

## Abstract

**Background:**

For holometabolous insects there has been an explosion of proteomic and peptidomic information thanks to large genome sequencing projects. Heterometabolous insects, although comprising many important species, have been far less studied. The migratory locust *Locusta migratoria*, a heterometabolous insect, is one of the most infamous agricultural pests. They undergo a well-known and profound phase transition from the relatively harmless solitary form to a ferocious gregarious form. The underlying regulatory mechanisms of this phase transition are not fully understood, but it is undoubtedly that neuropeptides are involved. However, neuropeptide research in locusts is hampered by the absence of genomic information.

**Results:**

Recently, EST (Expressed Sequence Tag) databases from *Locusta migratoria *were constructed. Using bioinformatical tools, we searched these EST databases specifically for neuropeptide precursors. Based on known locust neuropeptide sequences, we confirmed the sequence of several previously identified neuropeptide precursors (i.e. pacifastin-related peptides), which consolidated our method. In addition, we found two novel neuroparsin precursors and annotated the hitherto unknown tachykinin precursor. Besides one of the known tachykinin peptides, this EST contained an additional tachykinin-like sequence. Using neuropeptide precursors from *Drosophila melanogaster *as a query, we succeeded in annotating the *Locusta *neuropeptide F, allatostatin-C and ecdysis-triggering hormone precursor, which until now had not been identified in locusts or in any other heterometabolous insect. For the tachykinin precursor, the ecdysis-triggering hormone precursor and the allatostatin-C precursor, translation of the predicted neuropeptides in neural tissues was confirmed with mass spectrometric techniques.

**Conclusion:**

In this study we describe the annotation of 6 novel neuropeptide precursors and the neuropeptides they encode from the migratory locust, *Locusta migratoria*. By combining the manual annotation of neuropeptides with experimental evidence provided by mass spectrometry, we demonstrate that the genes are not only transcribed but also translated into precursor proteins. In addition, we show which neuropeptides are cleaved from these precursor proteins and how they are post-translationally modified.

## Background

The desert locust (*Schistocerca gregaria*) and the African migratory locust (*Locusta migratoria*) are insects with a well-recognised human impact and ecological and economical importance. They show the interesting phenomenon of phase polymorphism. In the harmless solitarious phase their population density is limited to a few individuals per hectare. Under certain circumstances, as a result of external stimuli and internal (endocrinological) changes, solitarious locusts can switch to gregarious behaviour and begin to aggregate, marching in 'hopper bands' and swarming as adults. This gregarious form destroys all vegetation with a devastating effect on the environment and economy, and has a severe impact on the residents of the afflicted regions (from acute allergic responses to famine). The neuro-hormonal mechanisms that drive and accompany this transition are still far from completely understood, however, it has been proven that several classical neurotransmitters/modulators [1], pheromones [2] and also neuropeptides [3,4], play an important role. The latter is not surprising, as (neuro)peptides are the largest class of signalling molecules found in animals. They can act as a transmitter, modulator and/or hormone and are known to be involved in most, if not all, physiological processes in Metazoa. Neuropeptides originate from larger precursor proteins. These are processed in the endoplasmatic reticulum to produce the actual bioactive peptides, ranging in size from a few amino acids to around 100. Processing of neuropeptides in insects generally occurs at the dibasic sites KR, RR or KK or at a single R residue proceeded by a basic amino acid residue at position -4, -6 or -8 [5]. In many cases, one neuropeptide precursor contains multiple neuropeptide isoforms and/or related peptides often containing a consensus motif. Some precursors produce structurally related peptides possessing equivalent potencies, whereas others have been shown to yield peptides with different (occasionally opposite) activities [6]. Therefore, it is important to identify all peptides originating from a single precursor since each of these peptides might have a different physiological relevance.

A large number of neuropeptides has already been identified and characterised in locusts, however, only for a few peptides the precursor protein is known. In the first instance (eighties and early nineties) peptide identification was done by means of laborious extraction procedures in combination with numerous bioassays and purification steps (for review see [7]). At a given point the steady stream of newly bioactive neuropeptides stagnated due to the restrictions of the available bioassays. With the introduction of "peptidomics" at the start of the new millennium, neuropeptide research was boosted once again. With the combination of new mass spectrometric methods, genomic databases and bioinformatic tools, it became possible to identify new peptides without the necessity to sacrifice vast amounts of animals. This led to an explosion in the number of identified neuropeptides in for example *D. melanogaster *[8] and *Caenorhabditis elegans *[9]. Also in the locust, this new peptidomics technique led to the identification of several new neuropeptides [10,11] and in addition dozens of partial sequences (unpublished results). The mass spectral analysis mostly generates a small sequence tag (a few amino acids), which is then compared with a genomic database to yield the full sequence. Unfortunately, for locusts very little genome sequence information is available and as the size of their genome is unusually large (it is expected to be up to 30 times larger than the genome of *D. melanogaster *[12]), no sequencing efforts are likely to be undertaken. This is a major pitfall for neuropeptide identification in locusts and the search for their presumed role in locust phase transition. In general, compared to the genome sequencing projects in the group of Holometabola, genome information in heterometabolous insects is scarce, hindering peptidome identifications.

Recently, an EST database from *L. migratoria*, comprising 76 012 raw EST data which were assembled into 12 161 clustered unigenes, was constructed and deposited in GenBank [13,14]. Only 30% of the genes discovered were annotated based on sequence similarity with other species, among which only one group of (neuro)peptide genes (pacifastin-like sequences). Neuropeptides, because of their small size and very limited similarity (mostly the conserved active core comprises only a few amino acids), are often neglected in annotation studies. In this study, we thoroughly searched the locust EST database specifically for the presence of neuropeptide precursors using various bioinformatic tools bearing in mind the typical features of a neuropeptide precursor.

## Results and discussion

In the present study, we examined the EST database of *L. migratoria *generated by Kang et al. [13] for sequences that encode putative neuropeptides and their respective precursors. Besides a whole body database (from gregarious fifth instar locusts), ESTs were generated from the head, hind legs and midgut from solitarious and gregarious locusts separately.

In first instance, we performed BLAST searches using all known neuropeptide precursors from *L. migratoria *and a related species, *S. gregaria*, as queries. In a second search, neuropeptides from *L. migratoria*, for which the precursor proteins are not known, were used as an input query in the BLAST searches. In this analysis, neuropeptide sequences that are expected to originate from a single precursor were combined (all possible combinations) and each neuropeptide sequence was flanked by typical processing sites [(G)KR, (G)(R)R or (G)KK]. As a third group of queries, neuropeptide precursors from *D. melanogaster *were used for screening the locust EST database.

The resulting EST sequences were further analysed to identify start and stop codons and typical neuropeptide precursor features (length, possible signal peptide (SignalP), cleavage sites, post-translational modifications). This way, we found several new *Locusta *neuropeptide precursors, which are discussed below. This strategy also provided interesting and valuable information concerning the link between heterometabolous and holometabolous insects at the peptide level.

### Pacifastins

The pacifastins are a family of low molecular weight serine protease inhibitors from arthropods. They display sequence similarities with nine cysteine rich domains in the light chain of pacifastin, a heterodimeric serine protease inhibitor isolated from the hemolymph of the crayfish *Pacifastacus leniusculus*, hence their name pacifastins [15]. They are characterised by a conserved pattern of six cysteine residues **Cys**-Xaa_9–12_-**Cys**-Asn-Xaa-**Cys**-Xaa-**Cys**-Xaa_2–3_-Gly-Xaa_3–6_-**Cys**-Thr-Xaa_3_-**Cys**. They were first discovered in locusts, but *in silico *data mining of expressed sequence tags databases revealed the existence of additional pacifastin-like polypeptides in Lepidoptera, Diptera, Coleoptera, and Siphonaptera [16].

Thus far, 10 pacifastin-related precursors encoding together 22 different peptides have been characterised in *L. migratoria *and *S. gregaria *[16-18]]. In *L. migratoria *3 pacifastin-related precursors were cloned and sequenced (LMPP 1–3) [19,20]. Blasting the LMPP sequences against the EST databases resulted in more than 140 hits (E-values ranging from e^-112 ^to e^-4^), signifying the presence of pacifastin mRNAs in whole-body, midgut, hind leg and head. The presence of pacifastin mRNA in the head provides additional evidence that the brain is one of the sites of pacifastin biosynthesis. Although pacifastin peptides had been shown in several brain areas before, so far there was always controversy whether or not this was due to contamination by the hemolymph, where the pacifastins are known to be present in high concentrations [21,22]. Most of the hits represent different length ESTs corresponding to one of the three previously cloned LMPP precursor genes (LMPP 1–3) [19,20], which again shows the high abundance of these pacifastins in locusts. These findings are a good indication that the predefined parameters are chosen well and allow an adequate analysis.

LMPP-1 contains two protease inhibitor peptides (LMPI-1, also called PMP-D2 and LMPI-2, also called PMP-C), that were both isolated and sequenced before [21]. LMPP-2 displays three potential peptide sequences, one of which was isolated before (LMPI-3, previously named HI or hemolymph inhibitor) [23]. The LMPP-3 gene encodes for four putative additional protease inhibitor peptides, none of which was isolated before.

**Figure 1:**
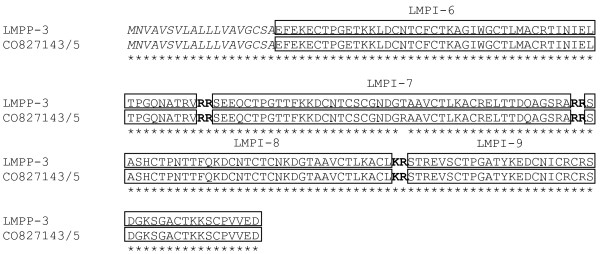
***Locusta migratoria *Pacifastin Precursor 3**. Amino acid sequence alignment of LMPP-3 and the ORF of CO827143 and CO827145 (most identical sequences). The signal peptide is indicated in italic, dibasic cleavage sites are shown in bold and pacifastin-like peptides are marked. Identical residues are marked with an asterisk.

Only the ESTs corresponding to the LMPP-3 gene differ from the previously cloned LMPP-3 gene in one amino acid within the LMPI-7 peptide, as a result of one different nucleotide (AGA coding for R in ESTs instead of ACA coding for T in cDNA cloning). As none of the sequenced LMPP-3 ESTs shows a threonine residue at that position, we can assume that SEEQCTPGTTFKKDCNTCSCGNDG**R**AAVCTLKACRELTTDQAGSRA is the correct sequence for LMPI-7 (Fig. 1). It cannot be excluded that the long list of hits also includes ESTs corresponding to hitherto unknown *Locusta *pacifastin precursors.

Based on the number of pacifastin ESTs that were found in both phases, the pacifastin genes are predicted to be differentially expressed in the solitary and gregarious phase with a higher prevalence in solitarious locusts [13]. This was previously also shown in a peptide differential display study by Clynen et al. [4].

### Neuroparsins

The neuroparsins are a family of structurally related peptides from arthropods (crustaceans, insects, chelicerates) that is characterised by a pattern of positional conserved cysteine residues and shows striking similarities to the vertebrate insulin-like growth factor binding proteins (IGFBP). The neuroparsins were first discovered in *L. migratoria*, where they are produced by neurosecretory cells in the pars intercerebralis region of the brain and then transported to the corpora cardiaca, where they are stored prior to release into the hemolymph [24]. They are multifunctional neurohormones displaying antijuvenile [25], antidiuretic [26], hyperglycemic, hyperlipemic [27] and neuritogenic effects [28]. In *L. migratoria *one neuroparsin precursor has been cloned and sequenced [29]. From this precursor several N-terminally truncated polypeptide isoforms (neuroparsin-A 1–4 and neuroparsin-B) originate.

In the desert locust *S. gregaria *4 different neuroparsin precursor cDNAs (Scg-NPP 1–4) were cloned and sequenced [30,31]. While NPP-1 and 2 are restricted to the brain, NPP-3 and 4 also occur in peripheral tissue (fat body, reproductive system). All 4 neuroparsin precursors are identical in the N-terminal region (first 61 amino acid residues) and contain at least 10 cysteine residues. Recently, evidence was provided for a phase-dependent transcriptional regulation of neuroparsin-encoding genes [32,33]. It was shown that neuroparsin transcripts are generally more abundant in solitarious locusts. In addition, in contrast to the gregarious phase, solitarious animals also contain detectable transcript levels of NPP 1–2 in the fat body.

A BLAST search with the *L. migratoria *neuroparsin precursor resulted in 5 hits with E-value < 5e^-26^. Three hits (CO849956, CO849957 and CO849958) were derived from the solitarious phase head cDNA library, one from the gregarious head (CO832876) and one from the gregarious whole-body (CO821291) cDNA library.

**Figure 2:**
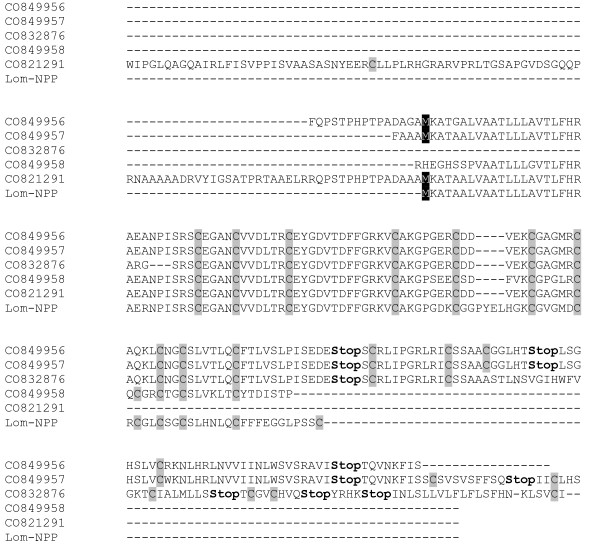
***Locusta migratoria *Neuroparsin Precursors**. Multiple sequence alignment (ClustalW 1.82) of CO849956, CO849957, CO832876, CO849958, CO821291 and the known *Locusta *neuroparsin precursor (Lom-NPP). Translation initiation sites are indicated in black and cysteine residues are shaded.

None of these hits, however, gave an exact match, meaning that they represent novel *Locusta *neuroparsin-like sequences (Fig. 2). This is not surprising, as also in the desert locust 4 different neuroparsin precursor cDNAs (Scg-NPP 1–4) are present.

Four of the EST tags (CO849956, CO849957, CO832876 and CO821291) most likely represent the same gene, as their protein-coding sequence is identical except for one amino acid in the signal peptide (A versus G in CO849956 as a result of one different nucleotide), which is probably due to a sequencing error. This novel neuroparsin-like sequence in *L. migratoria *shows 83% identity with NPP-4 of *S. gregaria*, both containing 10 cysteine residues.

The protein-coding sequence of CO849958 differs substantially from the other ESTs. Although both the start and stop codon is missing, this EST clearly represents a member of the neuroparsin family. The lack of a start codon is probably due to a sequencing error at the beginning of the EST, while at the 3' end the protein-coding sequence is incomplete. The partial sequence displays 76% identity with NPP-2 from *S. gregaria*. An attempt to complete the 3' end by the contig assembly procedure failed. Blasting the *Schistocerca *neuroparsin precursors against the *Locusta *EST database yielded no additional significant hits.

### Tachykinins

The tachykinins belong to an evolutionary conserved family of peptide neurotransmitters that play major roles in signalling in the nervous system and intestine of both vertebrates and invertebrates. The invertebrate tachykinin family is characterised by a common C-terminal sequence -FXGXRamide (X being a variable amino acid residue) analogous to the vertebrate consensus -FXGLMamide. The first members of the invertebrate tachykinin family were isolated as myotropic peptides from the central nervous system of *L. migratoria *[34,35]. All four *Locusta *tachykinins contain the C-terminal -FXGVRamide (X being Y or H) sequence. Their precursor protein was as yet unknown.

**Figure 3:**
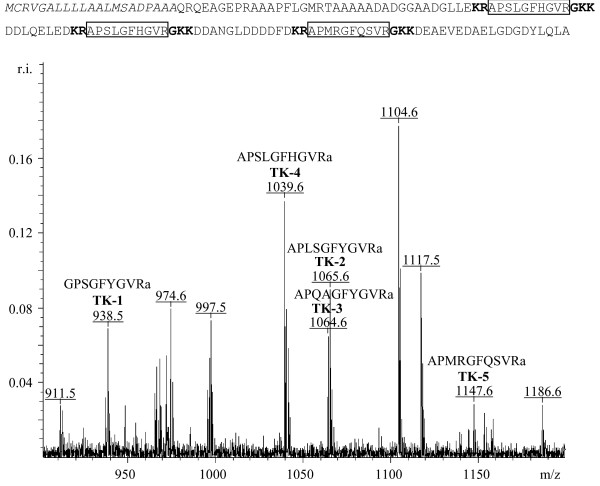
***Locusta migratoria *Tachykinin Precursor (top) and MS spectrum displaying the monoisotopic masses of 5 different Tachykinins (bottom)**. Amino acid sequence corresponding to the ORF of CO848687. The signal peptide as predicted by SignalP is shown in italic, possible amidation and dibasic cleavage sites are indicated in bold, possible tachykinin-like peptides are marked. MALDI-TOF mass spectrum of the first abdominal ganglion of *Locusta migratoria*. Masses corresponding to tachykinin peptides are marked (r.i. is relative intensity, m/z is mass to charge ratio).

When blasting the four tachykinin sequences separately, only tachykinin-4 gave one hit with a gregarious phase midgut cDNA sequence (CO848687), though with a relatively high E-value (0.13). Blasting the four tachykinin peptides in line (separated by GKR) against the EST database gave the same hit with a lower E-value 1e^-7^. This clearly demonstrates the benefit of combining several peptides that are expected to originate from the same precursor. The same hit was found when blasting the *D. melanogaster *tachykinin precursor (1e^-4^). The translated protein has all typical features of a neuropeptide precursor: SignalP predicts the presence of a signal peptide and several possible dibasic cleavage sites are present (Fig. 3). The EST is incomplete at the 3' side, as no stop codon is present. Of the four previously characterised *Locusta *tachykinin peptides, only TK-4 gives a perfect match and is present twice in this *Locusta *tachykinin precursor. The sequences of TK 1–3 were not found within this precursor, meaning that either there is more than one tachykinin precursor present in *L. migratoria*, or TK 1–3 are encoded by the missing part of the precursor at the 3' end. The latter assumption is strengthened by the fact that in all invertebrate species investigated so far, a single tachykinin precursor gene encodes multiple tachykinin forms [36]. The *D. melanogaster *genome contains a single tachykinin precursor, coding for six putative tachykinin sequences, five of which were identified in a peptide extract of *Drosophila *central nervous systems [37]. Also in cockroaches, up to 15 tachykinins are encoded by the same precursor gene [38]. A second argument is that the novel (partial) *Locusta *tachykinin precursor is only 141 amino acid residues in length, while all known insect tachykinin precursors vary between 289 and 366 amino acid residues.

Besides Lom-TK-4, the *Locusta *tachykinin precursor contains another putative tachykinin-like peptide (APMRGFQSVRamide), separated by dibasic cleavage sites. The C-terminus of this peptide resembles the -FXGXRamide consensus sequence for invertebrate tachykinins, except for the G, which is replaced by S. The mass of this putative peptide was calculated (1146.6 Da) and we searched MALDI-TOF mass spectra from the entire *Locusta *neuroendocrine system for the presence of this peptide. The exact mass was found in peptide extracts from the brain (proto-, deutero- and tritocerebrum), circumoesophageal connectives, suboesophageal ganglion, frontal ganglion, hypocerebral ganglion, pro-, meso and metathoracic ganglion and all five abdominal ganglia (Fig. 3). The novel tachykinin mass was neither found in the corpora allata nor in the neurohemal organs of the head (corpora cardiaca) and the abdomen (perisympathetic organs). The exact masses of the other four known tachykinins (TK 1–4) were found in the same peptide extracts. The fact that the distribution of the novel tachykinin is identical to that of the other four *Locusta *tachykinins presents an additional argument that all these *Locusta *tachykinins most likely originate from the same precursor. The presence of the precursor in the midgut (midgut EST library) is consistent with earlier immunocytochemical studies and the purification of a tachykinin from the midgut of *S. gregaria *[39,40].

To confirm that the 1146.6 Da peptide found in the MALDI-spectra genuinely corresponds to the tachykinin-like peptide, we analysed a peptide extract of 10 ventral nerve cords by ESI-Q-TOF MSMS and fragmented the ion corresponding to the 1146.6 Da peptide. Hereby we confirmed its sequence. This proves that this peptide is produced from the tachykinin precursor through a common endoproteolytic pathway as seen for most neuropeptides. Hence, this peptide was designated as tachykinin-5 (TK-5). The presence of a peptide with an aberrant C-terminus it not unusual, as also in *Leucophaea maderae*, *Periplaneta americana, Apis mellifera *and *D. melanogaster *tachykinins with a substitution in the C-terminal consensus sequence occur. In both cockroach species it was proven that these peptides (-FXAXRamides) are also translated *in vivo *by demonstrating their presence in tissue extracts by mass spectrometry [38].

### Ecdysis-triggering hormone

The ecdysis-triggering hormone (ETH) is one of the three principal neuroendocrine components known to regulate ecdysis behaviour, together with eclosion hormone (EH) and crustacean cardioactive peptide (CCAP). To date, ETHs have only been identified in the lepidopterans *Manduca sexta *and *Bombyx mori *and in the dipterans *D. melanogaster *and *Anopheles gambiae *[41-45]]. The ETH-genes encode two peptides with ETH-like structure and biological activity: ETH and pre-ETH in Lepidoptera and ETH-1 and 2 in Diptera.

**Figure 4:**
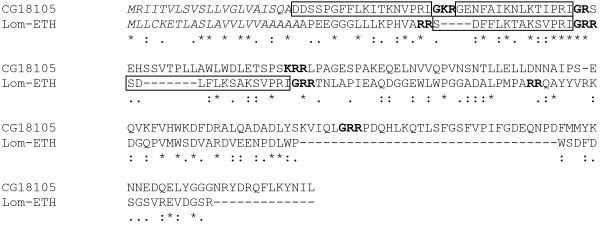
***Locusta migratoria *Ecdysis Triggering Hormone Precursor. **Amino acid sequence alignment of CG18105 (the *Drosophila *ETH-precursor) and the novel *Locusta *ETH-precursor (assembly of ORF from CO822155 and CO833211). The signal peptide is indicated in italic, amidation sites and dibasic cleavage sites are marked in bold and (putative) ETH-peptides are marked.

Blasting the *D. melanogaster *ETH precursor against the *L. migratoria *EST database gave one significant hit (E-value 6e^-6^) in the gregarious phase whole body cDNA library (CO822155). There are several indications that this EST actually corresponds to the *L. migratoria *ETH precursor: the translated protein has all features of a typical neuropeptide precursor, displaying a signal sequence and several dibasic cleavage sites. In addition, the precursor contains two peptides having a C-terminal -PRIamide, which is a typical consensus sequence for the ETH peptides (-PRVamide in Mas-PETH and Bom-PETH, -PRMamide in Mas-ETH and Bom-ETH, -PRIamide in Drm-ETH-2 and Aga-ETH-1 and 2 and -PRLamide in Drm-ETH-1). The *Locusta *EST sequence does not contain a stop codon. However, blasting the nucleotide sequence of CO822155 against the EST database gave two hits with an overlap in the C-terminal region (CO833211 and CO822156). This way we were able to complete the EST precursor sequence (Fig. 4).

**Figure 5:**
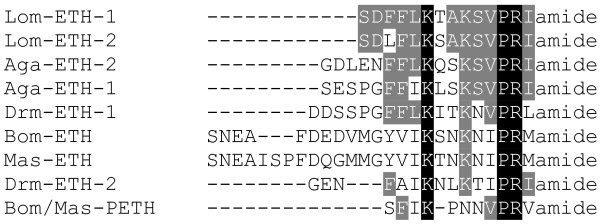
Amino acid sequence alignment of all known ETH-like peptides.

The two putative *Locusta *ETH-peptides were designated as Lom-ETH-1 (SDFFLKTAKSVPRIamide) and Lom-ETH-2 (SDLFLKSAKSVPRIamide). They show most sequence similarity with the *Anopheles *ETHs (Fig. 5), sharing the C-terminal KSVPRIamide sequence. This EST provides the first biochemical evidence for the presence of ETHs in a heterometabolous insect.

**Figure 6:**
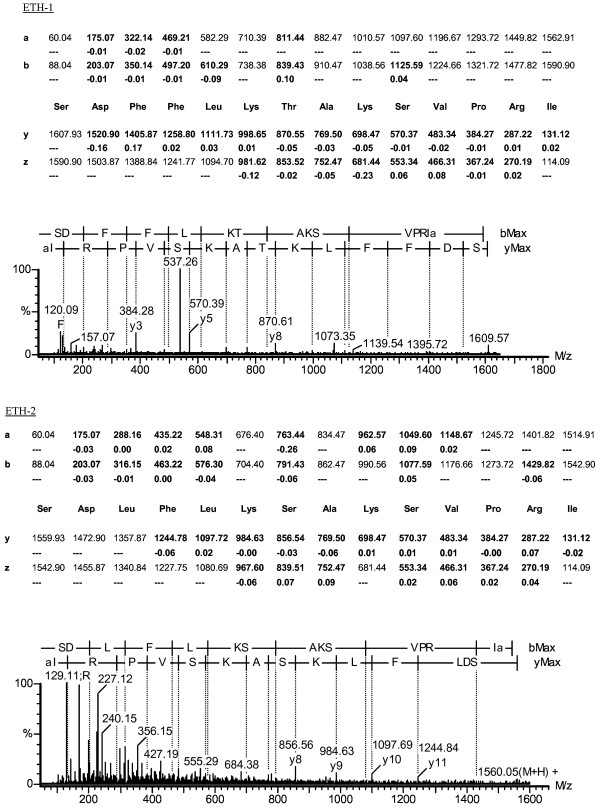
**MSMS spectra of the two locust ETH-peptides**. MSMS fragmentation spectrum of the triple charged ion at m/z 536.67, corresponding to ETH-1 (SDFFLKTAKSVPRIamide) and the double charged ion at m/z 780.45, corresponding to ETH-2 (SDLFLKSAKSVPRIamide). A-type, b-type, y-type and z-type fragment ions are shown. The theoretical fragment ion masses found in the spectrum are indicated in bold and the mass difference between the expected and observed fragment ion masses is shown below.

The ETHs are produced by the epitracheal organs, which are distributed along the tracheae and mainly consist of Inka cells. In *D. melanogaster*, the Inka cells are referred to as peritracheal cells. The peritracheal cell system is widely conserved in insects. Several holometabolous and heterometabolous insect orders show PETH-immunoreactivity among peritracheal cells [42]. The morphology and distribution along the tracheae varies considerably. In Orthoptera, tracheae have numerous immunopositive cells scattered on both the major and minor branches. We dissected tracheae from fifth instar *L. migratoria *just before adult ecdysis and analysed a peptide extract of these tracheae by nanoLC-Q-TOF MS(MS). Both the predicted masses of Lom-ETH-1 and Lom-ETH-2 (respectively 1606.9 Da and 1558.9 Da) were present and their sequences were confirmed by MSMS fragmentation analysis (Fig. 6), proving that these mature ETH-peptides are processed from the ETH-precursor.

### Neuropeptide F

Neuropeptide Y, the most abundant neuropeptide in the mammalian nervous system, is a highly conserved 36 amino acid neuromodulator [46]. The invertebrate NPY-related peptides are divided into two groups based on their size: the shortNPFs ranging in size from 8 to 11 amino acid residues and the (long)NPFs ranging in size from 36 to 40 amino acid residues. The longNPFs are considered as the invertebrate homologues of vertebrate NPY. Whereas the vertebrate NPYs C-terminally end with an amidated tyrosine (Y) residue, all NPFs end with an amidated phenylalanine (F) residue. The NPFs have been found in platyhelminths [47,48], molluscs [49,50] and insects [51]. In *D. melanogaster*, both the *short NPF *(*sNPF*; CG13968) and the (*long*) *Drosophila NPF *(*dNPF*; CG10342) gene have been characterised. *dNPF *regulates larval feeding behaviour [52]. The *dNPF *gene is highly expressed in larvae attracted to food and is turned off in older larvae that exhibit food aversion, hypermobility (wandering stage) and co-operative burrowing. Overexpression of the *dNPF *gene in older larvae prolonged feeding whereas down regulation in young larvae induced food aversion. Also *sNPF *was found to control food intake and regulate body size [53]. These findings suggest a functional similarity with vertebrates as NPY also exerts a central role in the regulation of feeding behaviour [54].

**Figure 7:**
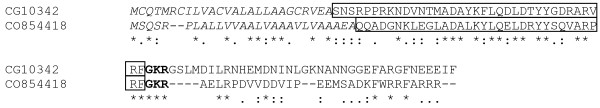
***Locusta migratoria *Neuropeptide F**. Amino acid sequence alignment of CG10342 (the *Drosophila *NPF-precursor) and the ORF of CO854418. The signal peptide is indicated in italic, possible amidation and dibasic cleavage sites are marked in bold and a (putative) NPF-peptide is marked.

In locusts, recently two shortNPF peptides were characterised (unpublished results). Until now, nothing was known about longNPF in locusts, or in any other heterometabolous insect. Blasting the dNPF precursor against the *L. migratoria *EST database gave one significant hit (E-value 7e^-6^) with the solitarious phase head cDNA library (CO854418). There are several indications that this EST in fact corresponds to the *L. migratoria *longNPF precursor: the translated protein has all features of a typical neuropeptide precursor, displaying a signal sequence immediately followed by the NPF sequence (33 amino acid residues) with the typical -RXRFamide C-terminal motif, an amidation signal and a dibasic cleavage site (Fig. 7). The presence of this long neuropeptide F in the locust nervous system could not be demonstrated by mass spectrometry as yet.

### Allatostatin-C

**Figure 8:**
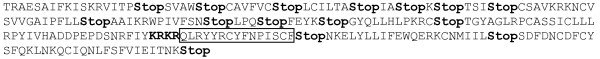
***Locusta migratoria *Allatostatin-C**. Amino acid sequence corresponding to the ORF of CO835369. Possible dibasic cleavage sites and translation stops are indicated in bold, a possible allatostatin-C peptide is marked.

The allatostatins were originally isolated from brain tissue as inhibitors of juvenile hormone biosynthesis by the corpora allata, hence their name allatostatins [55]. After the discovery of additional allatostatins in a variety of insect orders they were classified in three different peptide groups based on their sequence similarity. There is the large group of the allatostatin A-type that have the common Y/FXFGL/Iamide C-terminal sequence, the allatostatin B-type which share a -WX_6_Wamide C-terminal sequence and the allatostatin C-Type with a common -PISCF C-terminal sequence. Besides the allatostatic activity of these three groups, which is confined to a specific insect order (Dictyoptera), they all appear to be pleiotropic in function [56,57]. Thus far, only in *D. melanogaster *the three genes that encode the three different types of allatostatins have been identified [58-60]]. Blasting with the *D. melanogaster *allatostatin precursors resulted in a good similarity for allatostatin-C with an EST sequence from gregarious head cDNA (CO835369). Although the E-value turned out to be rather high (4e^-4^) and the translated sequence yielded a number of stop codons, the peptide sequence itself is very good preserved (Q**V**RY**RQ**CYFNPISCF in *D. melanogaster *and Q**L**RY**YR**CYFNPISCF in *L. migratoria*) (Fig. 8). Also the Lepidopteran allatostatin-C (Q**V**R**FRQ**CYFNPISCF), the only other allatostatin-C identified thus far, is almost identical. This is the first report of an allatostatin-C sequence in locusts and in heterometabolous insects in general. The allatostatin B-type was discovered as a myoinhibiting peptide in *L. migratoria *[61], whereas several A-type allatostatins have been purified and sequenced in *S. gregaria*, for which the precursor has also been cloned and sequenced [62,63]. The presence of all three types of allatostatins in a representative of the insect order Diptera (*D. melanogaster*) and Orthoptera (*L. migratoria*) strengthens the assumption that all insects (holometabolous and heterometabolous) have peptides belonging to the three different allatostatin peptide families. The mass corresponding to this novel allatostatin-C peptide displaying one disulphide bridge (1969.9 Da) was found in peptide extracts of the ventral nerve cord, indicating that this mature peptide is processed from the presumed allatostatin-C precursor.

## Conclusion

Heretofore 9 neuropeptide precursors had been identified in *L. migratoria*, including the pacifastin-like precursor 1–3, adipokinetic hormone precursor 1–3, insulin-related peptide precursor, neuroparsin precursor and ion transport peptide precursor. In this study this number was increased to 15, as we annotated the peptide precursor transcripts of two novel neuroparsin precursors, the tachykinin precursor, ecdysis-triggering hormone precursor, long neuropeptide F precursor and allatostatin-C precursor in the EST database published by Kang et al [13]. These annotations were based on a profound sequence homology with neuropeptide precursors that have been cloned and sequenced previously in locusts (pacifastins and neuroparsins) [19,20] or, for the smaller peptides, were supported by mass spectrometry data from this study (tachykinins, ecdysis-triggering hormones and allatostatin-C). Our search method was validated by the detection of three pacifastin-like precursors that were previously cloned and sequenced [19,20]. Although blasting the neuroparsin peptide precursor did not result in an exact match, it did reveal the presence of two novel neuroparsin-related precursors. These novel precursors can be considered as paralogues, which probably evolved differently after a gene duplication event in evolution. Both newly annotated neuroparsin transcripts show high similarity with previously identified neuroparsin precursors from *S. gregaria*.

The BLAST search program is not optimal for detecting small peptides. In fact, no specialised search tools for small peptides are available to date. To circumvent this problem, we combined several peptide isoforms and (post-translational) processing sites in a single search. This way, the hitherto unknown *Locusta *tachykinin precursor was identified, which not only contained two copies of a known tachykinin peptide (TK-4), but in addition a putative novel tachykinin-related peptide (TK-5). Mass spectrometrical analyses of peptide fractions from the *Locusta *central nervous system revealed that this peptide is actually generated as an endogenous peptide via post-translational cleavage at the dibasic cleavage sites. Although the other 3 known tachykinins were not contained within this newly predicted precursor, the absence of a stop codon leaves open the possibility that the 3 other known tachykinin sequences may be located more C-terminally within this same precursor.

Blasting neuropeptide precursors from *D. melanogaster *also proved to be a successful approach. This way, we were able to annotate the long neuropeptide F, ecdysis-triggering hormone, and allatostatin-C precursor orthologues in *L. migratoria*. This is the first time that these peptide precursors have been demonstrated in a heterometabolous insect. *L. migratoria *(heterometabolous) and *D. melanogaster *(holometabolous) have followed different evolutionary paths, meaning that these peptides have been well conserved during evolution.

Several other neuropeptide(precursors) were not found within our EST database search. It is possible that these are not present in the EST database (which does not cover the entire transcriptome) or that these neuropeptides are not encoded at all in the genome of *L. migratoria*. We also have to keep in mind that our analysis of the EST database is biased towards some factors. It is estimated that 3% of ESTs contain sequencing errors. Because of these errors, translated sequences could be wrong or insignificant at first sight. A misread nucleotide can cause the formation of a stop codon or a missing nucleotide can disturb the reading frame.

This study is a nice example of how bioinformatics can offer a solution for biological questions. New precursors and peptides are now found in a relatively easy manner, compared with the months and years sometimes needed for a biologist to characterise just one peptide precursor. In the future these EST data can be used to design primers for cloning and full-length sequencing of the respective genes. The characterisation of the precursor protein can lead to the characterisation of novel peptide isoforms encoded by the same precursor protein (see tachykinin). Moreover, comparison of precursor sequences enables us to obtain more crucial information about the evolutionary and/or interphyletic relationship than does comparison of peptide sequences alone. Comparable studies have been conducted for holometabolous insects (*D. melanogaster *and *Anopheles gambiae*), however, here genomic data were mined to annotate neuropeptide precursors instead of EST data [64-67]]. Our study is also unique in that respect that for three neuropeptide precursors our data were consolidated by mass spectrometry.

## Methods

### Database searches

All presently known neuropeptide (precursor) sequences from *L. migratoria*,*S. gregaria *and *D. melanogaster *were used in a homology search with the recently generated EST databases of *L. migratoria*, deposited in the GenBank database under the accession nos. CO819675–CO832059 and CO832067–C0865130[13]. For this purpose, tblastn, a program of the BLAST family, was used. The database was set to EST_others and was limited to the *Locusta *EST databases created by Kang et al. By entering the accession numbers under entrez query. For all other parameters default values were used. Signal peptide cleavage sites were predicted by using SignalP 3.0 [68]. Putative neuropeptide precursors were aligned by using CLUSTALW [69].

### Animals and peptide extraction

*L. migratoria *was raised under laboratory conditions [70], under a 13 h light, 11 h dark photoperiod at a constant temperature of 32°C and relative humidity between 40 and 60%. They were kept under gregarious conditions in cages of 38 cm × 38 cm × 38 cm that contained at least 100 locusts and fed daily with fresh grass and oatmeal *ad libitum*. Both male and female locusts were decapitated without previous anaesthesia. Neural tissues (brain parts, suboesophageal ganglia, circumoesophageal connectives, corpora cardiaca, corpora allata, frontal ganglia, hypocerebral ganglia, thoracic ganglia, abdominal ganglia, abdominal perisympathetic organs) from adult locusts (5 equivalents) and tracheae from fifth instar larvae (approximately 15 equivalents) were dissected in Ringer solution and transferred to a 0.5-ml Eppendorf tube on ice, containing 50 μl methanol/water/acetic acid (90/9/1) for neural tissues and 200 μl of the same solution for tracheae (Ringer solution, 9.82 g/l NaCl, 0.32 g/l CaCl_2, _0.48 g/l KCl, 0.73 g/l MgCl_2_, 0.25 g/l NaHCO_3_, 0.19 g/l NaH_2_PO_4_, pH 6.5). The samples were sonicated (on ice) three times for 1 min and the remaining solid fraction was centrifuged down (10 min at 9500 *g*).

Prior to the MS analysis the samples (neural tissues) were concentrated and desalted using ZipTipC_18 _pipette tips (Millipore, 15 μm). For this purpose, the supernatants were dried in a vacuum centrifuge and reconstituted in 50 μl of 0.1% aqueous TFA. The ZipTipC_18 _was pre-equilibrated for sample binding using 0.1% aqueous TFA containing 50% CH_3_CN, followed by 0.1% aqueous TFA. The sample was loaded onto the ZipTipC_18 _and, after flushing with 0.1% aqueous TFA to remove salts and other impurities, eluted in 4 μl 0.1% aqueous formic acid (FA) containing 70% CH_3_CN.

For nanoLC-Q-TOF MS(MS) (tracheae), the supernatant was filtered through a spindown filter (Ultrafree-MC, 0.22 μm, Millipore). The filtrate was dried in a vacuum centrifuge (Speedvac Concentrator SVC200H, Savant) and subsequently redissolved in 0.1% aqueous FA.

### Mass spectrometry

Matrix-assisted laser desorption/ionisation (MALDI) time-of-flight (TOF) mass spectrometry was performed on a Reflex IV (Bruker Daltonic GmbH), equipped with a N_2 _laser and pulsed ion extraction accessory. One μl of the sample solution was transferred to a ground steel target plate, mixed with 0.5 μl of a saturated solution of α-cyano-4-hydroxycinnamic acid in acetone and air-dried. The instrument was calibrated using a standard peptide mixture (Bruker Daltonic GmbH). Spectra were recorded in the reflectron mode within a mass range from m/z 500 to m/z 3000.

Nanoflow electrospray ionisation (ESI) double quadrupole (Qq) orthogonal acceleration (oa) time-of-flight (TOF) mass spectrometry was performed on a Q-tof instrument (Micromass, UK). One microliter of the sample solution was loaded into a gold-coated capillary (Long NanoES spray capillaries for Micromass Q-tof, Protana Engineering A/S). The sample was sprayed at a typical flow rate of 30 nl/min giving extended analysis time in which MS spectra as well as several MSMS spectra were acquired. During MSMS or tandem mass spectrometry, fragment ions are generated from a selected precursor ion by collision-induced dissociation.

Nanoflow liquid chromatography (nanoLC) Q-TOF MS(MS) was conducted using a Famos autosampler, a Switchos column-switching device and an Ultimate HPLC pump (all LC Packings) coupled to a Q-TOF mass spectrometer (Micromass) as described earlier [8]. Ten μl of sample (corresponding to approximately 15 tracheae) was loaded. The separation was done on a NanoEase Atlantis™ Dc18 column (3 μm, 100 μm × 100 mm, Waters) using a linear gradient from 95% solvent A, 5% solvent B to 40% solvent A, 60% solvent B in 50 minutes, with a constant flow rate of 150 nl/min (solvent A: 0.1% FA in water; solvent B: 0.1% FA in CH_3_CN). The outlet of the HPLC was connected to the electrospray interface of the Q-TOF mass spectrometer. The column eluent was directed through a stainless steel nano-bore emitter (Proxeon). Tandem mass spectrometry was conduced in an automated fashion. Doubly and triply charged ions above a certain threshold were selected for fragmentation by collision-induced dissociation. The applied collision energy was chosen automatically depending on the number of charges and the mass to charge ratio of the selected ion.

## Abbreviations

EST: Expressed Sequence Tag

*D. melanogaster*: *Drosophila melanogaster*

*L. migratoria*: *Locusta migratoria*

BLAST: Basic Local Alignment Search Tool

*S. gregaria*: *Schistocerca gregaria*

LMPP: *Locusta migratoria *Pacifastin-related Precursor

LMPI: *Locusta migratoria *Pacifastin-related Inhibitor

IGFBP: Insulin-like Growth Factor Binding Protein

Scg: *Schistocerca gregaria*

NPP: Neuroparsin Precursor

TK: Tachykinin

Lom: *Locusta migratoria*

MALDI: Matrix-Assisted Laser Desorption/Ionisation

ESI-Q: Electrospray Ionisation-Quadrupole

TOF: Time of Flight

MS: Mass Spectrometry

MSMS: Tandem Mass Spectrometry

ETH: Ecdysis-Triggering Hormone

EH: Eclosion Hormone

CCAP: Crustacean Cardioactive Peptide

Mas: *Manduca sexta*

PETH: Pre-Ecdysis-Triggering Hormone

Bom: *Bombyx mori*

Drm: *Drosophila melanogaster*

Aga: *Anopheles gambiae*

LC: Liquid Chromatography

NPY: Neuropeptide Y (C-terminal tyrosine residue)

NPF: Neuropeptide F (C-terminal phenylalanine residue)

sNPF: short Neuropeptide F

dNPF: *Drosophila *Neuropeptide F

TFA: trifluoroacetic acid

FA: formic acid

ORF: Open Reading Frame

## Authors' contributions

EC, JH and PV performed the *in silico *analyses and verified manually all significant BLAST hits prior to annotation. JH obtained the tissues for the MS(MS) surveys performed by EC. EC and JH drafted the manuscript. LS was involved in design and coordination of the study and revision of the manuscript. LS and ADL obtained funding for the study. All authors read and approved the final manuscript.
